# Author Correction: Acquisition, transmission and strain diversity of human gut-colonizing crAss-like phages

**DOI:** 10.1038/s41467-020-14599-0

**Published:** 2020-01-31

**Authors:** Benjamin A. Siranosian, Fiona B. Tamburini, Gavin Sherlock, Ami S. Bhatt

**Affiliations:** 1 Department of Genetics, Stanford University, Stanford, CA USA; 2 Department of Medicine, Division of Hematology, Stanford University, Stanford, CA USA

**Keywords:** Viral genetics, Bacteriophages, Clinical microbiology, Microbiome

Correction to: *Nature Communications* 10.1038/s41467-019-14103-3, published online 15 January 2020.

The original version of this Article contained an error in Fig. 1, in which the label ‘M1098 Infant 3M’ was inadvertently duplicated. This has been corrected in both the PDF and HTML versions of the Article.

