# Supplementary material for: Acquisition, transmission and strain diversity of human gut-colonizing crAss-like phages
Source: Nat Commun. 2020 Jan 15;11:280. doi: 10.1038/s41467-019-14103-3 (PMC6962324; doi:10.1038/s41467-019-14103-3)
Supplement: Supplementary file 1 — Supplementary Information [file 41467_2019_14103_MOESM1_ESM.pdf]

Acquisition, transmission and strain diversity of human gut-colonizing crAss-like phages

Supplementary Information

Siranosian et al.

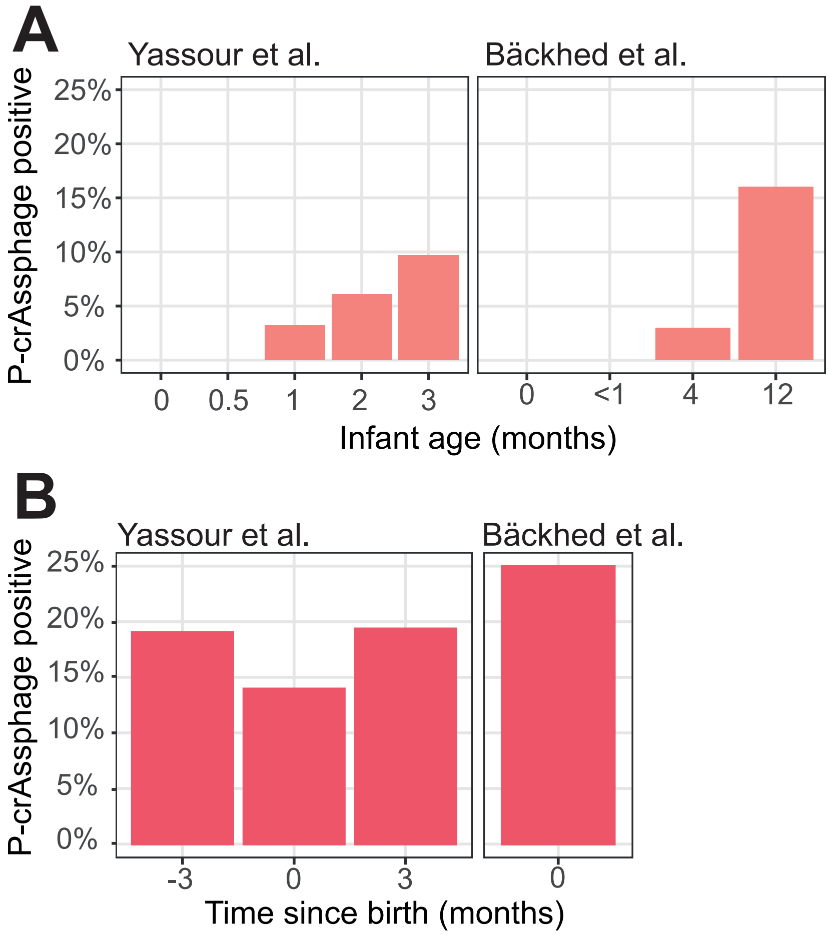

Supplementary Figure 1: P-crAssphage presence at 1x coverage in infant **(a)** and mother **(b)** samples.

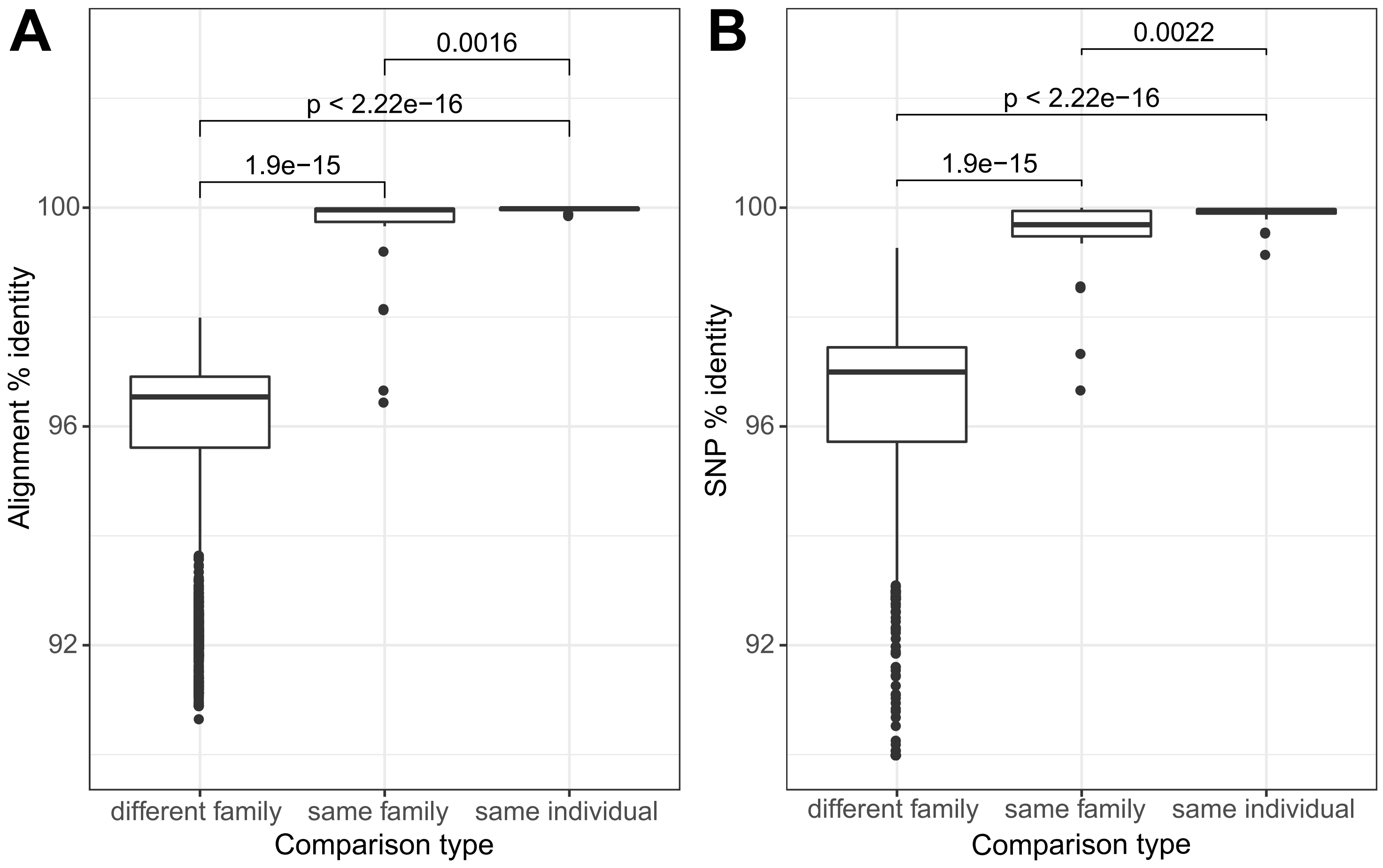

Supplementary Figure 2: P-crAssphage is more closely related in samples from mother-infant pairs than in samples from unrelated individuals. **a.** Distribution of pairwise alignment % identity of metagenome-assembled p-crAssphage genomes. Groups are separated by family relationships. P-values were calculated with the two-sided Wilcoxon rank sum test. **b.** Distribution of pairwise SNP % identity of p-crAssphage genomes. Groups are separated by family relationships. P-values were calculated with the two-sided Wilcoxon rank sum test. Boxes extend to the first and third quartile, whiskers extend to the upper and lower value within 1.5*IQR from the box. Outliers are shown as points.

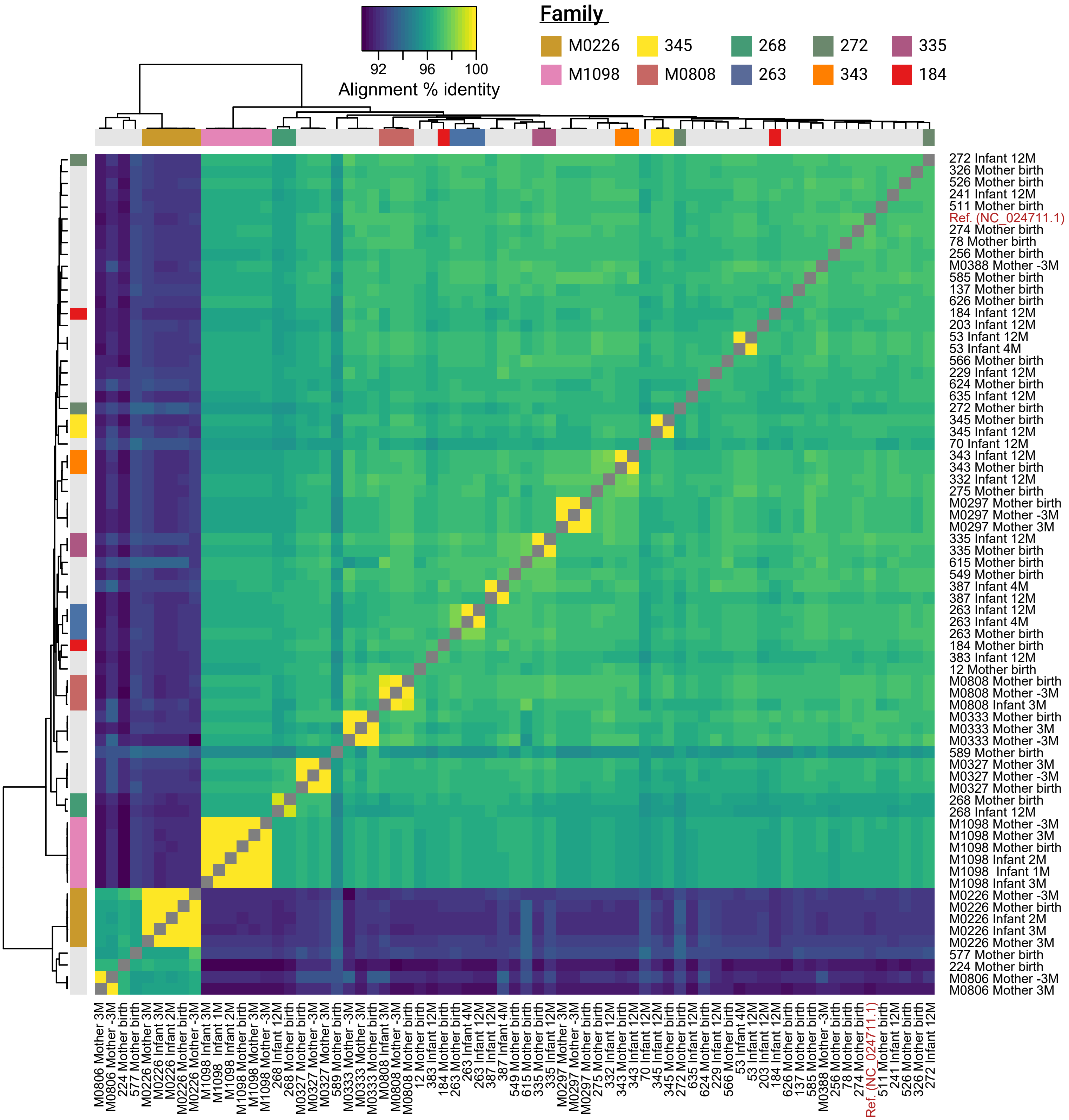

Supplementary Figure 3: Metagenome-assembled p-crAssphage genomes are highly similar in samples from matched mother-infant pairs. The heatmap shows pairwise alignment % identity in all mother-infant samples with p-crAssphage detected. The p-crAssphage reference genome is also included as a comparison.

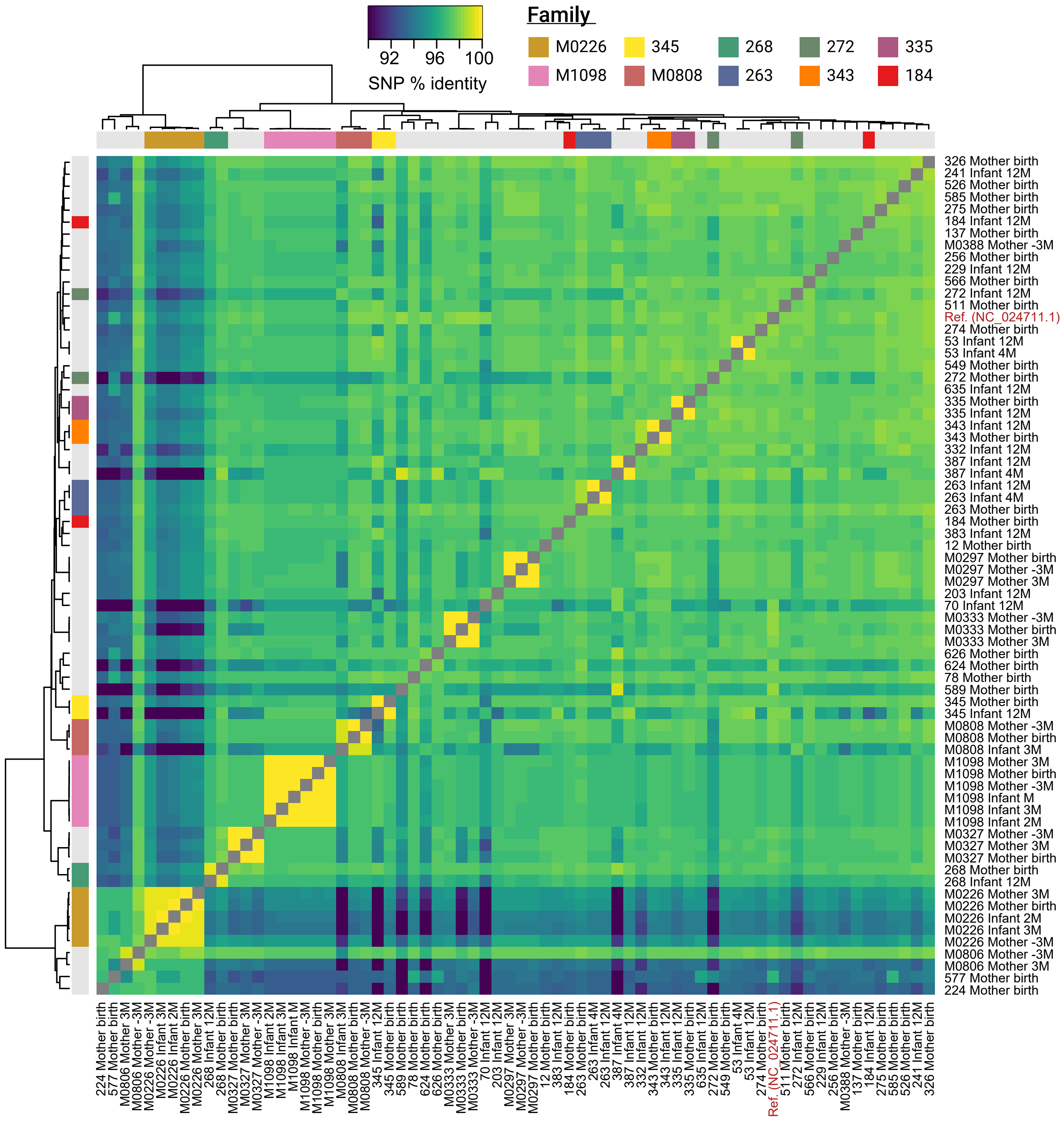

Supplementary Figure 4: P-crAssphage is highly similar at the SNP level in samples from matched mother-infant pairs. The heatmap shows pairwise SNP % identity in all samples with p-crAssphage detected.

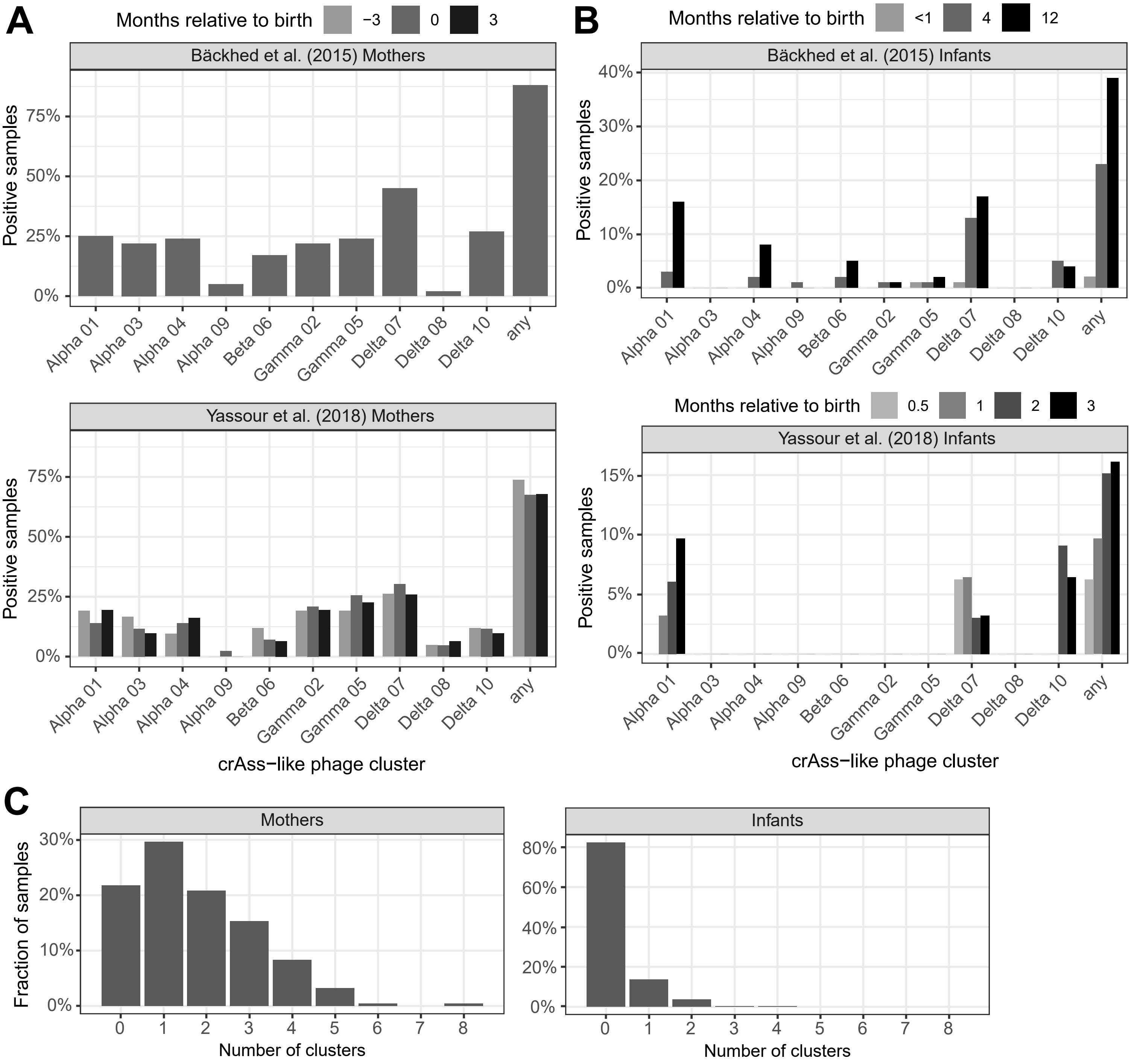

Supplementary Figure 5: CrAss-like phages (as defined in Guerin et al.[5]) detected at 1x covereage in mother and infant samples. **a.** CrAss-like phages detected in samples from mothers in each study. **b.** CrAss-like phages detected in samples from infants in each study. **c.** Number of crAss-like phage clusters detected in each sample from mothers and infants.

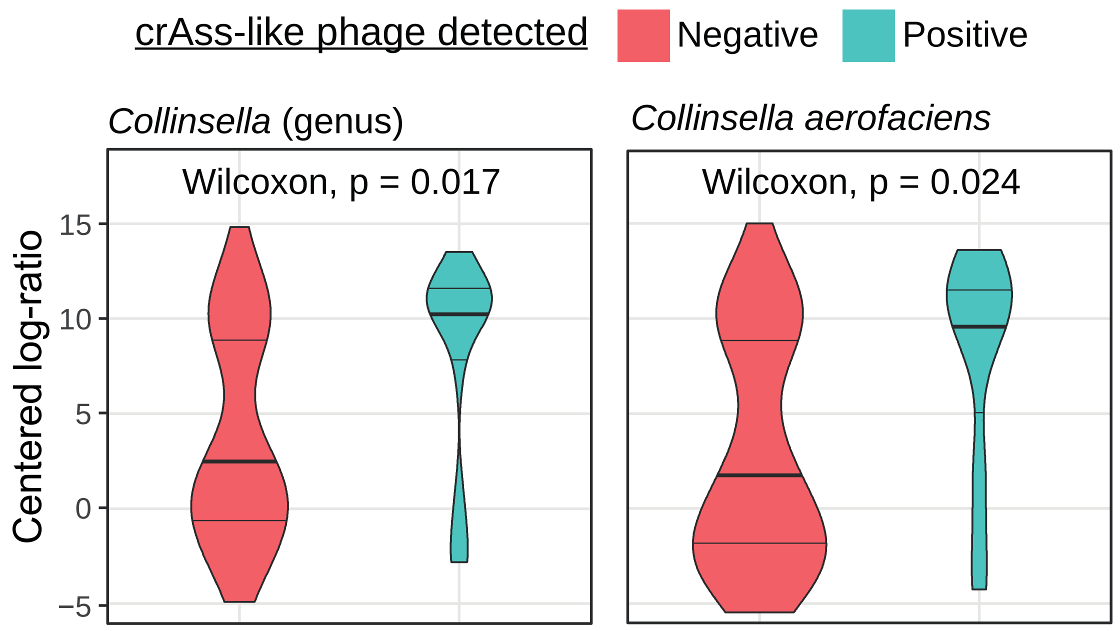

Supplementary Figure 6: Collinsella and Collinsella aerofaciens are at higher relative abundances in crAss-like phage positive vaginally delivered infants at 3-4 months of age, compared to crAss-like phage negative infants. P-values calculated with the two-sided Wilcoxon rank sum test and corrected for multiple hypothesis testing. Boxes extend to the first and third quartile, whiskers extend to the upper and lower value within 1.5*IQR from the box. Outliers are shown as points.

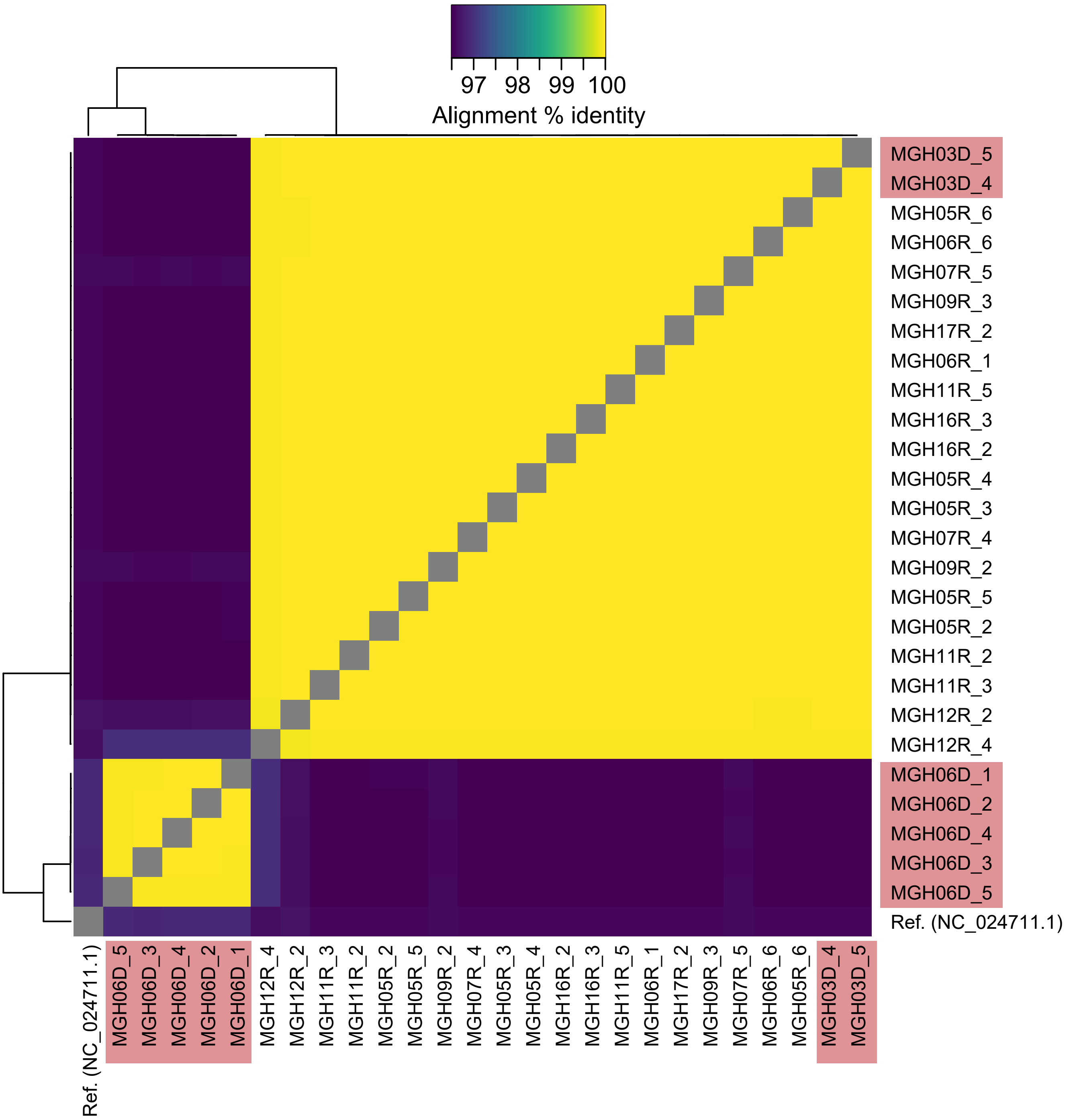

Supplementary Figure 7: Metagenome-assembled p-crAssphage genomes are highly similar in samples from matched FMT donor-recipient pairs in Smillie et al.[35]. The heatmap shows pairwise alignment % identity in all samples that assembled >50kb p-crAssphage sequence. Assembled genomes from donor samples are highlighted in red. The p-crAssphage reference genome is also included as a comparison.

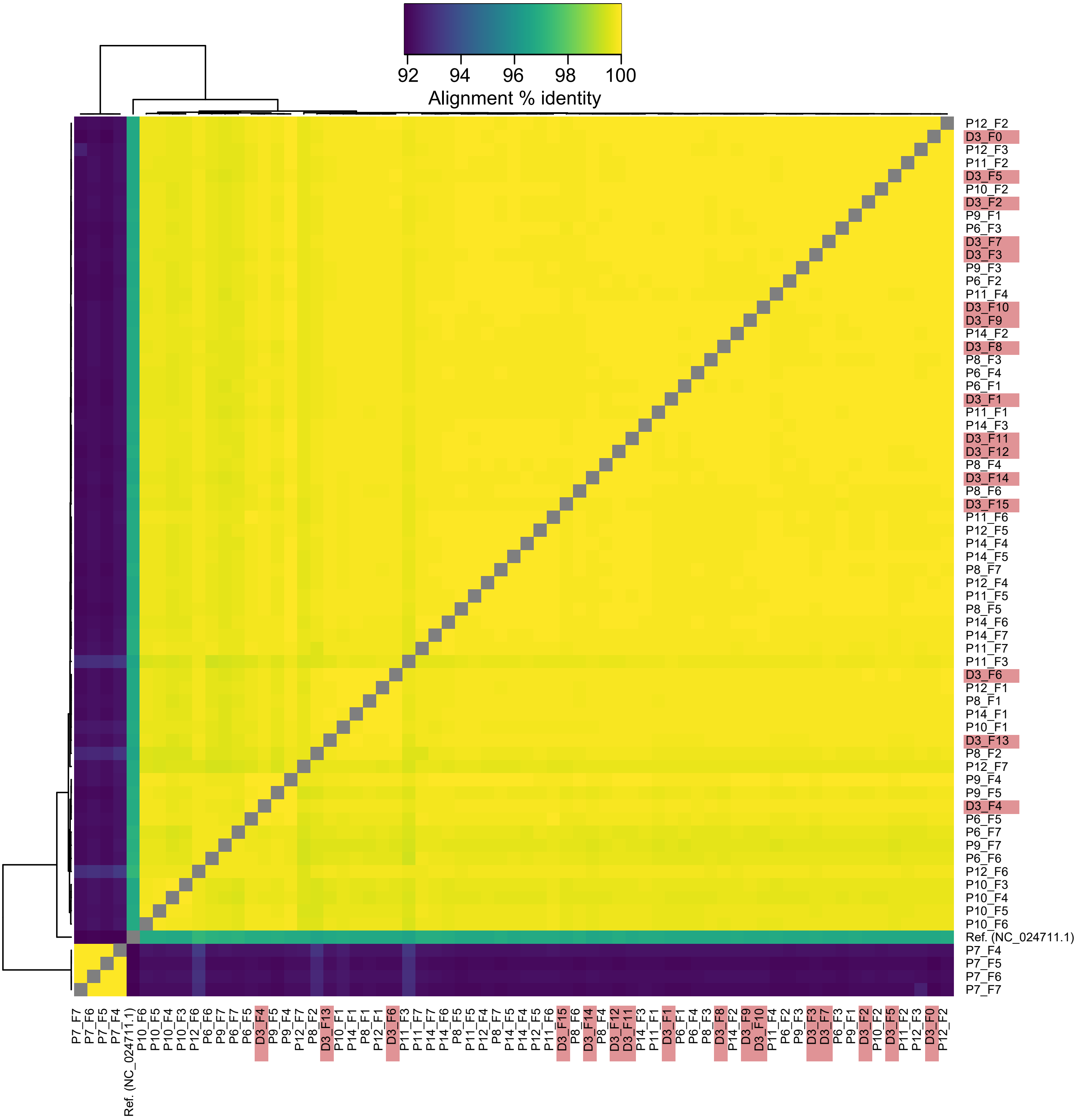

Supplementary Figure 8: Metagenome-assembled p-crAssphage genomes are highly similar in samples from matched FMT donor-recipient pairs in Draper et al.[37]. The heatmap shows pairwise alignment % identity in all samples that assembled >50kb p-crAssphage sequence. Assembled genomes from donor samples are highlighted in red. The p-crAssphage reference genome is also included as a comparison. The donor for patient P7 was p-crAssphage negative; this patient may have acquired their p-crAssphage from the environment or another source.

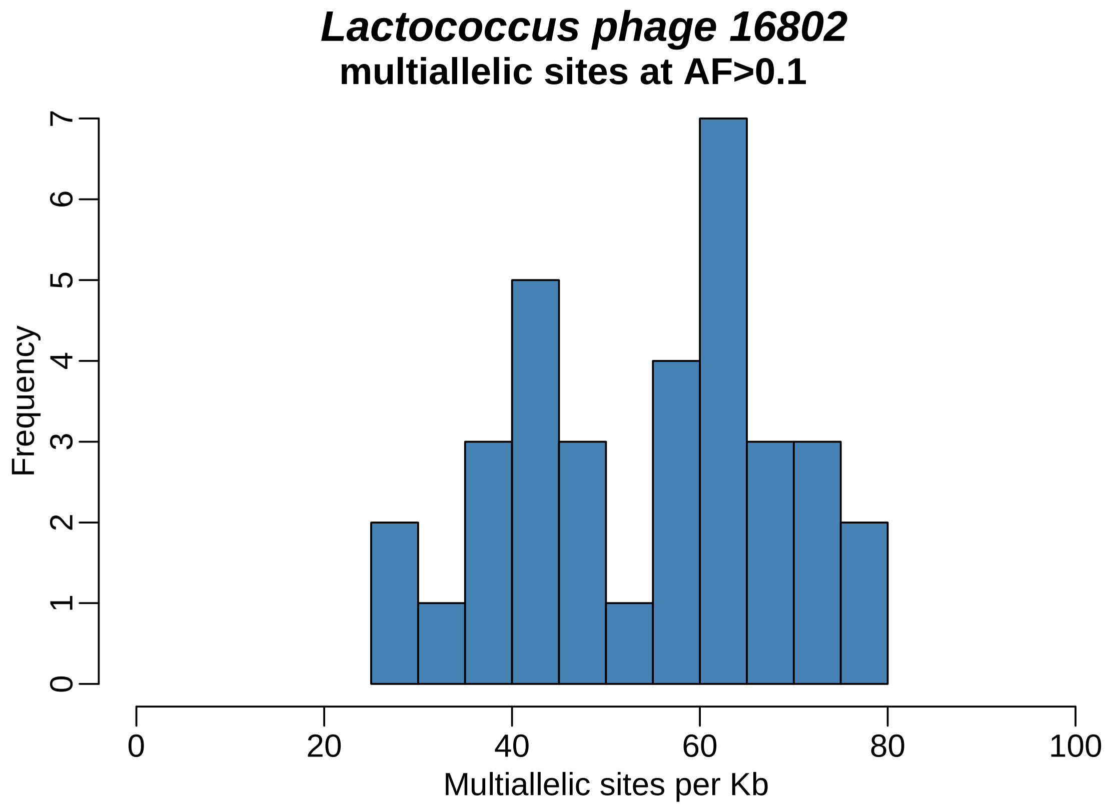

Supplementary Figure 9: Lactococcus phages were the only other group of phages detected with at least 1x coverage in at least ten mother and infant samples. This best represented member of this group, Lactococcus phage 16802, was detected in 34 samples and has more multiallelic sites than p-crAssphage on average, with a median of 58.8 multiallelic sites per kb.

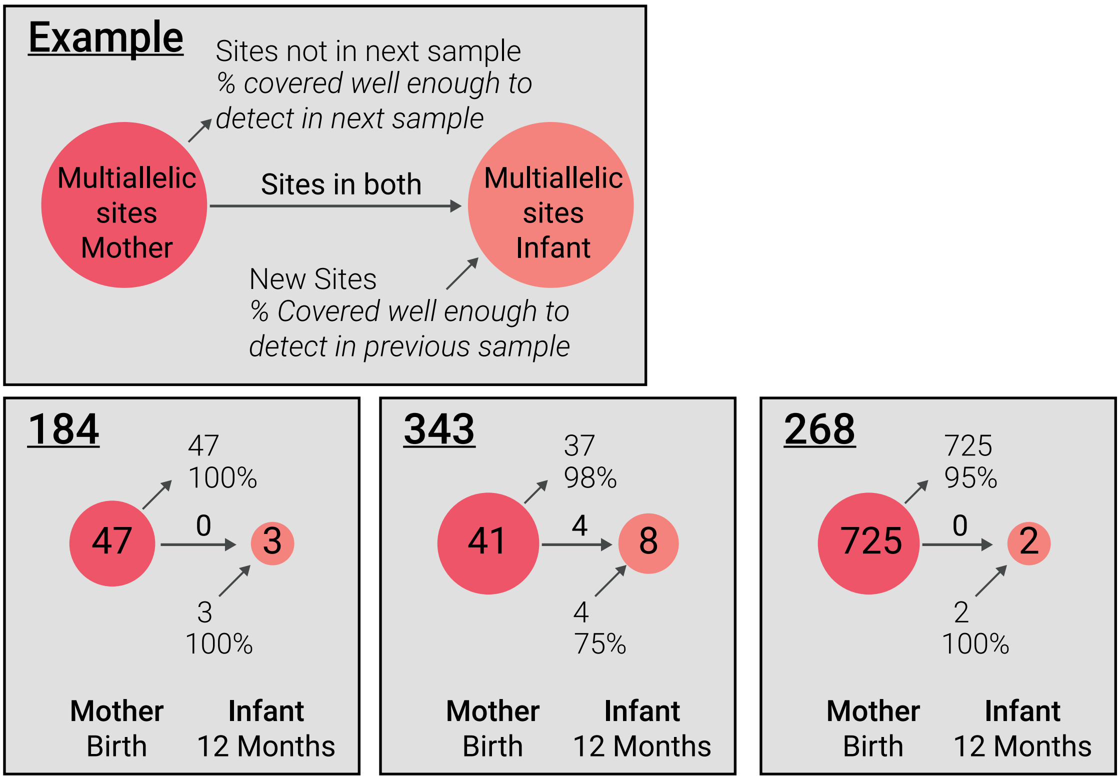

Supplementary Figure 10: Additional cases of multiallelic sites in mothers and infants with one p-crAssphage positive sample.
