# Supplementary material for: Acquisition, transmission and strain diversity of human gut-colonizing crAss-like phages
Source: Nat Commun. 2020 Jan 15;11:280. doi: 10.1038/s41467-019-14103-3 (PMC6962324; doi:10.1038/s41467-019-14103-3)
Supplement: Supplementary file 3 — Description of Additional Supplementary Files [file 41467_2019_14103_MOESM3_ESM.pdf]

Description of Additional Supplementary Files

File Name: Supplementary Data 1
Description:
Sheet 1) Sample and dataset metadata for mother-infant samples.
Sheet 2) Sample and dataset metadata for FMT samples.
Sheet 3) Associations between birth mode and crAss-like phage detection. Only comparisons with at least ten crAss-like phage positive infants across the two studies are shown. P-values were calculated with Fisher's exact test and are uncorrected for multiple hypothesis testing.

File Name: Supplementary Data 2
Description:
Sheet 1) Summary statistics of p-crAssphage metagenomic assembly in mother and infant samples.
Sheet 2) Individual sample statistics of p-crAssphage metagenomic assembly in mother and infant samples.

File Name: Supplementary Data 3
Description:
Sheet 1) Pairwise alignment comparisons between metagenomic-assembled p-crAssphage genomes in mother and infant samples.

File Name: Supplementary Data 4
Description:
Sheet 1) Multiallelic sites detected in p-crAssphage genes in samples from mothers.
Sheet 2) Multiallelic sites detected in p-crAssphage genes in samples from infants.

File Name: Supplementary Data 5
Description:
Sheet 1) Presence of crAss-like phage clusters in samples from mothers and infants.
Sheet 2) Summary statistics of crass-like phage metagenomic assembly in mother and infant samples.
Sheet 3) Individual sample statistics of crAss-like phage metagenomic assembly in mother and infant samples.
Sheet 4) Pairwise alignment comparisons between metagenomic-assembled crAss-like phage genomes in mother and infant samples.

File Name: Supplementary Data 6
Description:
Sheet 1) Table header explanation.
Sheet 2) Associations between crAss-like phage presence and bacterial relative abundances in vaginally born infants of 3-4 months of age.

File Name: Supplementary Data 7
Description:
P-crAssphage and crAss-like phage assembled genomes.
