# Supplementary material for: Acquisition, transmission and strain diversity of human gut-colonizing crAss-like phages
Source: Nat Commun. 2020 Jan 15;11:280. doi: 10.1038/s41467-019-14103-3 (PMC6962324; doi:10.1038/s41467-019-14103-3)
Supplement: Supplementary file 11 — Reporting Summary [file 41467_2019_14103_MOESM11_ESM.pdf]

nature research

Corresponding author(s): Ami Bhatt
Last updated by author(s): Dec 2, 2019

Reporting Summary

Nature Research wishes to improve the reproducibility of the work that we publish. This form provides structure for consistency and transparency in reporting. For further information on Nature Research policies, seeAuthors & Referees and theEditorial Policy Checklist .

Statistics

For all statistical analyses, confirm that the following items are present in the figure legend, table legend, main text, or Methods section.

n/a	Confirmed	
☐	☒	The exact sample size (*n*) for each experimental group/condition, given as a discrete number and unit of measurement
☐	☒	A statement on whether measurements were taken from distinct samples or whether the same sample was measured repeatedly
☐	☒	The statistical test(s) used AND whether they are one- or two-sided<br>*Only common tests should be described solely by name; describe more complex techniques in the Methods section.*
☐	☒	A description of all covariates tested
☐	☒	A description of any assumptions or corrections, such as tests of normality and adjustment for multiple comparisons
☐	☒	A full description of the statistical parameters including central tendency (e.g. means) or other basic estimates (e.g. regression coefficient) AND variation (e.g. standard deviation) or associated estimates of uncertainty (e.g. confidence intervals)
☐	☒	For null hypothesis testing, the test statistic (e.g. *F*, *t*, *r*) with confidence intervals, effect sizes, degrees of freedom and *P* value noted<br>*Give P values as exact values whenever suitable.*
☒	☐	For Bayesian analysis, information on the choice of priors and Markov chain Monte Carlo settings
☒	☐	For hierarchical and complex designs, identification of the appropriate level for tests and full reporting of outcomes
☒	☐	Estimates of effect sizes (e.g. Cohen's *d*, Pearson's *r*), indicating how they were calculated

Our web collection on statistics for biologists contains articles on many of the points above.

Software and code

Policy information about availability of computer code

Data collection	Data were download with NCBI fastq-dump version 2.9.0 (https://ncbi.github.io/sra-tools/fastq-dump.html)
Data analysis	TrimGalore version 0.5.043 was used to perform quality and adapter trimming<br>SeqKit version 0.9.144 was used to remove duplicates with the command<br>Reads were mapped against the human genome using BWA version 0.7.17-r1188<br>Kraken2 version 2.0.8_beta and Bracken version 2.5 were used for metagenomic classification<br>Preprocessed sequencing reads were assembled with SPAdes version 3.13.1<br>Pairwise comparisons were conducted with nucmer version 4.0.0beta2<br>SNPs were called with Snippy version 4.4.4<br>Filtering, decomposition and normalization of variants was conducted with vt version 0.552 and bcftools version 1.953.<br>Code for preprocessing metagenomic sequencing data available at https://github.com/bhattlab/bhattlab_workflows<br>Code for metagenomic classification with Kraken2 available at https://github.com/bhattlab/kraken2_classification<br>Code for pairwise comparison of assemblies and SNPs available at https://github.com/bhattlab/bhattlab_workflows

For manuscripts utilizing custom algorithms or software that are central to the research but not yet described in published literature, software must be made available to editors/reviewers. We strongly encourage code deposition in a community repository (e.g. GitHub). See the Nature Research guidelines for submitting code & software for further information.

Data

Policy information about availability of data

All manuscripts must include a data availability statement. This statement should provide the following information, where applicable:

- Accession codes, unique identifiers, or web links for publicly available datasets
- A list of figures that have associated raw data
- A description of any restrictions on data availability

All data available in this manuscript are publicly available. We have provided metagenome-assembled crAss-like phage genomes a supplementary file.

Field-specific reporting

Please select the one below that is the best fit for your research. If you are not sure, read the appropriate sections before making your selection.

☒ Life sciences ☐ Behavioural & social sciences ☐ Ecological, evolutionary & environmental sciences

For a reference copy of the document with all sections, see nature.com/documents/nr-reporting-summary-flat.pdf

Life sciences study design

All studies must disclose on these points even when the disclosure is negative.

Sample size	Sample size was determined by previously published data analyzed in this manuscript. Multiple studies with the same topic were analyzed to increase sample size.
Data exclusions	Metagenomic samples with fewer than 10,000 reads after preprocessing were discarded, as we were not able to accurately analyze crAss-like phages in samples with very low sequencing coverage.
Replication	As this manuscript analyzes publicly available data, no attempts at replication were made. We did analyze data from multiple studies with the same topic to confirm our findings.
Randomization	As this manuscript analyzes publicly available data, no randomization was used.
Blinding	Investigators were not blinded during data analysis.

Reporting for specific materials, systems and methods

We require information from authors about some types of materials, experimental systems and methods used in many studies. Here, indicate whether each material, system or method listed is relevant to your study. If you are not sure if a list item applies to your research, read the appropriate section before selecting a response.

Materials & experimental systems

n/a	Involved in the study
☒	☐ Antibodies
☒	☐ Eukaryotic cell lines
☒	☐ Palaeontology
☒	☐ Animals and other organisms
☒	☐ Human research participants
☒	☐ Clinical data

Methods

n/a	Involved in the study
☒	☐ ChIP-seq
☒	☐ Flow cytometry
☒	☐ MRI-based neuroimaging
